# Phenotypic Array for Identification and Screening of Antifungals against *Aspergillus* Isolates from Respiratory Infections in KwaZulu Natal, South Africa

**DOI:** 10.3390/jof9060616

**Published:** 2023-05-26

**Authors:** Sarla Naicker, Viresh Mohanlall, Sandile Ngubane, John Mellem, Nokuthula Peace Mchunu

**Affiliations:** 1 Department of Biotechnology and Food Science, Durban University of Technology, Durban 4000, Kwa-Zulu Natal, South Africa; sarlaj@dut.ac.za (S.N.); johnm@dut.ac.za (J.M.); 2 National Research Foundation, Pretoria 0001, Brummeria, South Africa

**Keywords:** *Aspergillus*, fungal infections, immunocompromised, resistance, azole, phenotypic array

## Abstract

The rapid emergence of invasive fungal infections correlates with the increasing population of immunocompromised individuals, with many cases leading to death. The progressive increase in the incidence of *Aspergillus* isolates is even more severe due to the clinical challenges in treating invasive infections in immunocompromised patients with respiratory conditions. Rapid detection and diagnosis are needed to reduce mortality in individuals with invasive aspergillosis-related infections and thus efficient identification impacts clinical success. The phenotypic array method was compared to conventional morphology and molecular identification on thirty-six *Aspergillus* species isolated from patients with respiratory infections at the Inkosi Albert Luthuli Hospital in Kwa-Zulu Natal. In addition, an antimicrobial array was also carried out to screen for possible novel antimicrobial compounds for treatment. Although traditional morphological techniques are useful, genetic identification was the most reliable, assigning 26 to *Aspergillus fumigatus* species, 8 *Aspergillus niger*, and 2 *Aspergillus flavus* including cryptic species of *A. niger*, *A. tubingensis and A. welwitschiae*. The phenotypic array technique was only able to identify isolates up to the genus level due to a lack of adequate reference clinical species in the database. However, this technique proved crucial in assessing a wide range of possible antimicrobial options after these isolates exhibited some resistance to azoles. Antifungal profiles of the thirty-six isolates on the routine azole voriconazole showed a resistance of 6%, with 61% having moderate susceptibility. All isolates resistant to the salvage therapy drug, posaconazole pose a serious concern. Significantly, *A. niger* was the only species resistant (25%) to voriconazole and has recently been reported as the species isolated from patients with COVID-19-associated pulmonary aspergillosis (CAPA). Phenotypic microarray showed that 83% of the isolates were susceptible to the 24 new compounds and novel compounds were identified for potentially effective combination treatment of fungal infections. This study also reports the first TR34/98 mutation in *Aspergillus* clinical isolates which is located in the *cyp51A* gene.

## 1. Introduction

The progressive increase in the incidence of *Aspergillus* isolates poses a challenge in treating invasive infections in immunocompromised patients [1]. There is a rapid emergence of invasive fungal infections (IFIs) with the increasing population of immunocompromised individuals. Fungal diseases such as invasive aspergillosis (IA) and candidiasis are the most frequently occurring infections in immunocompromised individuals [2]. The severity and impact of fungal infections have resulted in the World Health Organization drawing up the first-ever list of fungal pathogens that are potential threats to human health, divided into critical, high, and medium-priority categories, with *Aspergillus fumigatus* included in the critical group [3].

The rise in immune-deficient patients due to cancer, ageing, organ transplants, AIDS, and other invasive surgical procedures impacts the rising prevalence of infections. In resource-limiting settings common in African countries, invasive fungal infections remain understudied and underdiagnosed despite their high mortality rates compared with other infectious diseases [4]. More than 200,000 aspergilli-related infections are reported annually [5]. According to the Global AIDS Update in Geneva 2016 (HIV/AIDS 2019), fungal infections are frequent in South Africa and are mainly caused by the HIV/TB syndemic with this disease burden negatively influencing public health. Invasive fungal infections are also the major cause of HIV-related mortality and account for 50% of AIDS-related deaths globally [6]. In the study, [7] investigated the epidemiology of IA in an immunocompromised population (*n* = 563) and found that the lung tissue was the most frequent site of infection (94%) with *A. fumigatus* being the commonly isolated species (92%). This study also reported that IA was associated with high mortality and should be considered an emerging and devastating infectious disease in this population. The progressive increase in the incidence of *Aspergillus* isolates poses a clinical challenge in treating invasive infection in immunocompromised patients [1]. Therefore, there is a need for improved diagnostic and management procedures for fungal illnesses that can help reduce morbidity and mortality associated with these diseases [8].

Molecular techniques have emerged as standard approaches for microbiological infection [9]. However, in developing countries, cost and a lack of infrastructure continues to be a disadvantage. Thus, there is a need for early diagnosis to develop appropriate and productive antifungal therapy, which necessitates the use of rapid and effective identification tools. Novel phenotypic technology for rapid and specific identification has also been developed [10,11]. The Biolog advanced phenotypic technology (Biolog, Hayward, CA, USA) is one such technology that claims to provide phenotypic information on the properties of strains for species-level identification. The Biolog Identification System can rapidly identify species of fungi from the systems reference laboratory using proprietary redox chemistry. The Biolog identification for fungi is based on 95 discrete tests simultaneously which gives a characteristic reaction pattern called a “fingerprint”. This fingerprint reaction pattern provides a vast amount of information about the metabolic properties of each isolate tested, along with a species-level identification.

The rising incidence of invasive fungal infections has unfortunately coincided with a significant increase in azole resistance in *Aspergillus* [12]. Drug resistance in *Aspergillus* infections is catastrophic to these groups with chronic lung diseases or immunosuppression, which also has grown [13,14]. Effective antifungal therapy is crucial in treating the disease in patients with a compromised immune system, especially in cases of invasive pulmonary aspergillosis, which often advances very rapidly. Azole resistance in *Aspergillus* isolates further challenges this emergence because they are first-line therapies for aspergillosis [15]. However, the widespread proliferation of azole-resistant *A. fumigatus* isolates in medicine and the environment restricts antifungal therapy choices and may be linked to an elevated death rate [16].

Azole resistance is usually due to genetic alterations that may be stable or transitory in the genome of implicated strains [17]. Azoles are part of the class of compounds that target the 14-α sterol demethylase enzyme, also known as demethylation inhibitors (DMI) and are used not only in the clinical setting but also in the agricultural sector to control plant pathogens [18]. *Aspergillus* azole resistance, specifically, *A. fumigatus*, can be acquired through selective pressure (1) clinically during repeated and prolonged exposure to azole therapy or (2) via environmental exposure due to use in agriculture [19]. In both these likely situations, isolates with different resistance mechanisms and azole susceptibility profiles will be selected. However, the acquired resistance mechanism to azoles is likely due to mutations in the cyp51A gene [20]. The target molecule of azoles is *cpy51*, a lanosterol 14-∞—demethylase involved in the biosynthesis of ergosterol, essential fungal membrane lipid and ergosterol deficiency [21]. The most widely reported and frequent mutations in the coding sequence of the cyp51A gene that has led to azole resistance are on tandem-repeat (TR) insertions in the promoter region with mutations TR34 which usually co-occurs with a substitution of leucine to Histidine at position 98 (L98H) of the coding region [18]. There is another lower occurring insertion at the promoter region known as TR46. Isolates with these mutations can infect immunocompromised patients, limiting their treatment options and leading to serious disease progression and complications [22]. Although the number of cases of azole-resistant *A. fumigatus* recovered from clinical samples is still limited, azole resistance mechanisms continue to spread and increase globally, reducing the effectiveness of azole in aspergillosis treatment, thus, the increased mortality rate [13].

Fungal infections remain challenging for clinicians as the number of infections increases with the growing population of immunocompromised patients with an ever-increasing resistance to azole and limited therapeutic options [23]. Thus, this study aimed to determine the prevalence of *Aspergillus*species in respiratory infections in immunocompromised patients at the Inkosi Albert Luthuli Hospital in KwaZulu-Natal South Africa. Conventional morphological techniques were compared to molecular identification and phenotypic microarray was explored as a rapid and reliable alternative in diagnosis. Routine and salvage therapy azoles were assessed for susceptibility and the potential of new compounds for the effective treatment of *Aspergillus*infections was screened. In light of increasing drug resistance in azole monotherapy, this study also identified new compounds with low toxicity at therapeutic concentrations, for use in potentially effective combination treatment of fungal infections and thus increase the therapeutic range to reduce the resistance rate.

## 2. Materials and Methods

### 2.1. Sample Collection and Culture Maintenance

Thirty-six filamentous fungal samples previously isolated from the upper respiratory tract of patients at the Inkosi Albert Luthuli Hospital between July 2015 and June 2016 were collected and cultured. The isolates were sub-cultured and maintained on malt extract agar (MEA, Thermo Fisher Scientific, Waltham, MA, USA) and potato dextrose agar (PDA, Thermo Fisher Scientific, Waltham, MA, USA) plates.

### 2.2. Microscopic and Culture Morphology Identification

Conventional culture and microscopic techniques were used for preliminary identification. Ten microliters of the spore suspension were transferred onto MEA and incubated at 37 °C for 72 h and viewed under a light microscope for characteristic morphology. The plates were incubated further until sporulation and observed for expected growth, viz., and the color of spores.

### 2.3. DNA Identification

DNA was extracted from cultures first grown on MEA plates for 72 h at 37 °C, thereafter two plaques were transferred to 100 mL malt extract broth and incubated at 37 °C, 150 rpm for 18 h. The mycelia were centrifuged, and the pellet was used to extract DNA using the protocol by [24]with minor modifications.

A modified version of the protocol for the PCR was carried out [25,26]. The PCR assay was performed using 5 μL of the extracted DNA sample in a total reaction volume of 50 μL consisting of primers Forward primer 566: CAGCAGCCGCGGTAATTCC and Reverse 1200: CCCGTGTTGAGTCAAATTAAGC), 25 μL of Taq Readymix (Thermo Fisher Scientific, Waltham, MA, USA) for 30 cycles of DNA 95 °C for the 30 s, an annealing step at 58 °C for 30 s, and an extension step at 72 °C for 1 min, with a final extension at 72 °C for 5 min [25,26]. The PCR products were purified using DNA Purification Kit for sequencing (Qiagen, Germantown, MD, USA) following the manufacturer’s manual. A Thermo Scientific 1 kb Plus molecular marker was used. Sequencing of PCR samples was performed by Inqaba Biotech (South Africa) on a Sanger Sequencing Platform. Blast analysis was conducted for sequencing on National Centre Biotechnology Information (NCBI) (http://www.ncbi.nlm.nih.gov accessed on 15 March 2017). The sequences were aligned using Muscle 2 programme and the tree was done using Neighbor-Joining analysis and visualized on Treebase.

The following are the Genebank accession numbers for the isolates used in the study.

(OQ991322), (OQ991476), (OQ991320), (OQ991495), (OQ991474), (OQ997393), (OQ997391), (OQ99694), (OQ996947), (OQ996975), (OQ996937), (OQ996936), (OQ996935), (OQ996933), (OQ996932), (OQ996932), (OQ996930),(OQ996929), (OQ996885), (OQ996884), (OQ996883), (OQ996882), (OQ996877), (OQ996878), (OQ996885), (OQ996875), (OQ996874), (OQ996873), (OQ996872), (OQ996871).

### 2.4. Phenotypic Identification

The Biolog FF panel was used for the identification of filamentous fungi isolates. Each plate of the FF panels contains 96 wells (95 tests and one control, water) (Figure S1: Supplementary file). The isolates were inoculated (one plaque) on malt extract plates for 72 h at 37 °C. The spores were then harvested from the surface of the agar plates using a sterile swab by gently scraping off across the surface and transferred into the sterile inoculating fluid (FF-IF, Biolog, Hayward, CA, USA). The turbidity of the suspension was measured (Biolog turbidimeter) until it reached a density of approximately 75% transmittance, according to the manufacturer’s protocol (Biolog, Hayward, CA, USA). A hundred microliters of fungal inoculum were transferred into each well of the PM plates. Inoculated PM plates were incubated at 37 °C for 96 h. Data were collected at 12 h intervals, reading on the Microlog station at 750 nm.

### 2.5. Minimum Inhibitory Concentration (MIC) of Voriconazole and Posaconazole

The 36 *Aspergillus* isolates were tested against current therapeutic drugs, voriconazole and posaconazole, using the Liofilchem MIC Test Strip (MTS)(Figure S2: Supplementary file), with a concentration range of 0.002 to 32 μg/mL. Half a milliliter of spore suspensions (10^6^) was transferred and spread on malt extract agar plates. Azole MIC strips were then applied to the inoculated agar surface as per the manufacturer’s instructions (Liofilchem). Inhibition was assessed and recorded at 24 and 48 h to determine susceptibility and resistance to the azoles.

The Liofilchem MIC Test Strip uses a gradient test to determine the MIC of microorganisms to identify resistance patterns. The MIC test strips are impregnated with a predetermined concentration gradient of the antibiotic. When the MIC strip was applied to the inoculated agar surface, the pre-impregnated exponential gradient of the antimicrobial agent was immediately transferred to the agar matrix. After the appropriate incubation period, a symmetrical inhibition ellipse centered along the strip was formed. The MIC was read directly from the scale in terms of ug/mL at the point where the edge of the inhibition ellipse intersects the strip. The susceptibility of the isolates to the azoles was tested and analyzed as per the manufacturer’s protocol (Liofilchem MIC test strip mold, 2015). The isolates were scored as follows:

1. Voriconazole: MIC ≤ 0.25; intermediate susceptibility > 0.25–2.0; resistant > 2.0
2. Posaconazole: MIC ≤ 0.06; intermediate susceptibility > 0.06–0.25; resistant > 0.25

### 2.6. Screening of Antifungal Compounds Using Phenotypic Microarray

**Figure 1:**
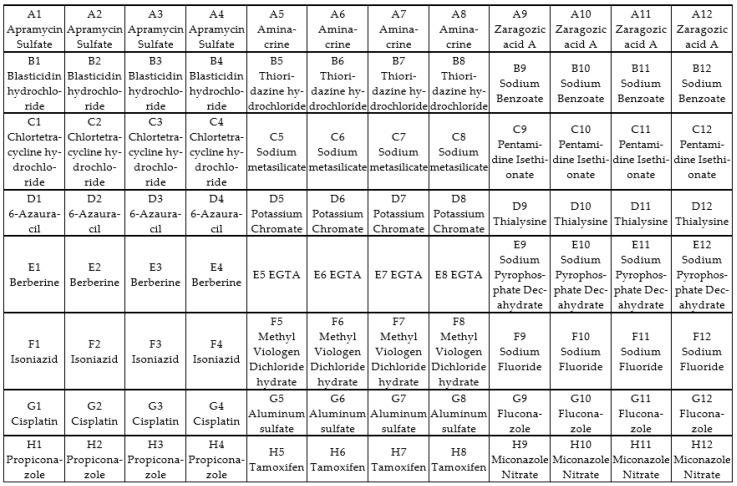
Chemical sensitivity panel PM 24C with 24 chemical agents in four concentrations.

An array plate containing 24 control compounds of varying concentrations was used to identify possible alternative chemicals as novel fungal control agents. Isolate preparation was performed as described in Section 2.4. The PM 24C panel was chosen as it contains compounds that can be used to control fungi. The 24 compounds in the Biolog chemical sensitivity panel vary from azoles, fungicides, antibiotics, and food preservatives to antimicrobial agents with varying concentrations (Figure 1).

The isolates were first inoculated on malt extract plates for 72 h at 37 °C for spore formation. The spores were then collected from the surface of the agar plates using a sterile swab by gently rubbing across the surface and then transferred into the sterile inoculating fluid (Biolog, Hayward, CA, USA). The turbidity of the suspension was measured using a Biolog turbidimeter and a density of approximately 75% transmittance was used as recommended by the manufacturer’s protocol. One hundred microliters of fungal inoculum were transferred into each well of the PM plates, followed by incubation at 37 °C for 96 h. Absorbance data (750 nm) were collected at 24 h intervals over 96 h, using the Microlog station to read the panels and capture data.

### 2.7. Identification of TR34/98 Mutations Associated with Azole Resistance

Cultures were grown on malt extract agar plates for three days at 37 °C. Two plaques were transferred to 100 mL malt extract broth and incubated at 37 °C, 150 rpm for 18 h. The mycelia were centrifuged, and the pellet was used to extract DNA. DNA was isolated as described in 2.3, followed by PCR amplification using specific primers (forward primers: P-A5-TCTCTGCACGCAAAGAAGAAC; CYP1-L-CACCCTCCCTGTGTCTCCT; CYP2-L-CATGTGCCACTTATTGAGAAGG; CYP3-L TTCCTCCGCTCCAGTACAAG) and (reverse primers: P-A7-TCATATGTTGCTCAGCGG; CYP1-R-AGCCTTGAAAGTTCGGTGAA; CYP2-R-CCTTGCGCATGATAGAGTGA; CYP3-RCCTTTGAAGTCCTCGATGGT) following the protocol explained in 2.3 [25,26], and then analyzed for associated genes to identify genetic markers for antifungal resistance. MUSCLE (multiple sequence comparison by log expectation) was used for multiple protein sequence alignment for the amino acid alignment of the wild-type (WT) cyp51A gene [27].

## 3. Results

### 3.1. Identification Using Traditional (Culture and Microscopy), Genetic and Phenotypic Methods

**Figure 2:**
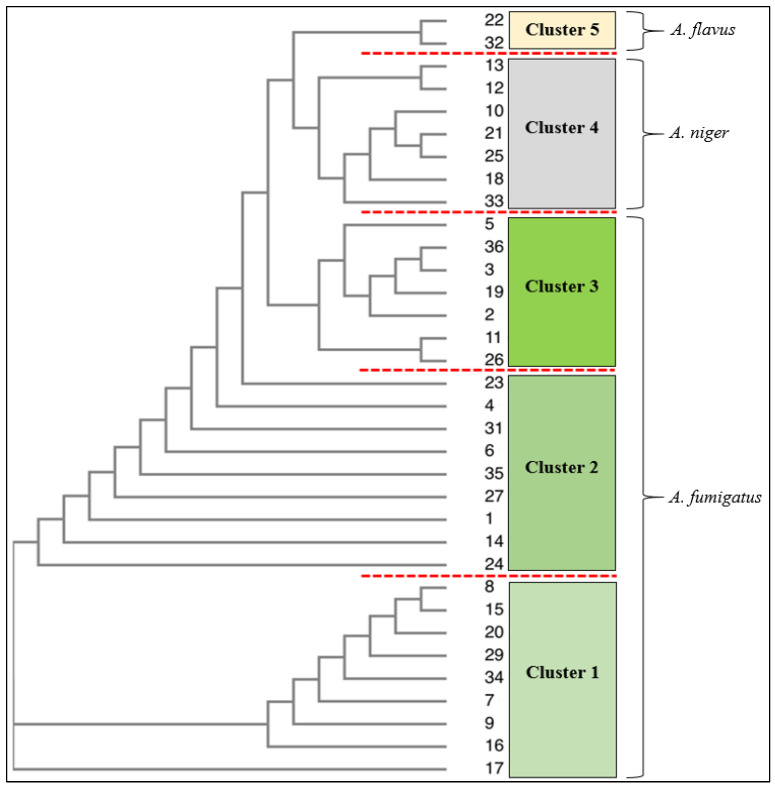
Phylogenic tree showing clusters within isolates of *Aspergillus species*. The sequences were aligned using Muscle 2 programme and the tree was done using Neighbor-Joining analysis and visualized on Treebase.

All thirty-six isolates from upper respiratory infections of immunocompromised patients were identified as *Aspergillus* species. Genetic testing using ITS confirmed twenty-six isolates to be *A. fumigatus*, eight *A. niger*, and two *A. flavus*. The results achieved by the conventional identification methods, viz. microscope and culture techniques, matched the identification confirmed by 18S rRNA; however, the culture methods were not able to distinguish cryptic species of *A. niger*, *A. tubingensis*, and *A. welwitschiae* which 18S rRNA achieved in isolates 12, 13, and 21 (Table 1). The phylogenetic tree further revealed that *A. niger* species (10, 21, 25, 18, 33) formed cluster 4, and isolates 12 and 13 (*A. niger/A. tubingensis*) form a sub-group within cluster 4 (Figure 2). The phylogenic tree with the supporting values is presented in the supplementary file (Figure S4). The Biolog identification system matched isolates to the genus level only. Seventy-two percent of the *Aspergillus* isolates from the respiratory infections were *A. fumigatus*, with 22% *A. niger* and 6% *A. flavus*. *A. fumigatus* was the most prevalent *Aspergillus* species isolated from upper respiratory infections.

Typical features of the respective *Aspergillus* species were observed to characterize and identify each species. The sporulation color was noted as a distinguishing cultural characteristic and conidia and mycelial forms were observed under the microscope to support the identification.

The Biolog system matches test isolates with the information in its database using biochemical profiles. The results of the Biolog are presented as a similarity (SM) value, of which 1.0 is a 100% match. Similarly, a “No ID” is given for similarity values of <0.5 and percentage match if between 0.5 and 0.9. None of the 36 isolates had a 100% match to the species level using the Biolog MicroStation Identification system (Table 1).Values between 0.5 and 0.9 provide a percentage identification of up to 99%. Identification by the Biolog system matched most of the isolates to the genus level only, in which the limitation could be the dataset present for biochemical matching. Ten of the 26 *A. fumigatus* isolates had similarity values of <0.1 and the remaining 16 have readings between 0.137 and 0.565, for a minimum of one and maximum of two species of the four possible matches.

### 3.2. Antifungal Analysis: Voriconazole and Posaconazole

**Figure 3:**
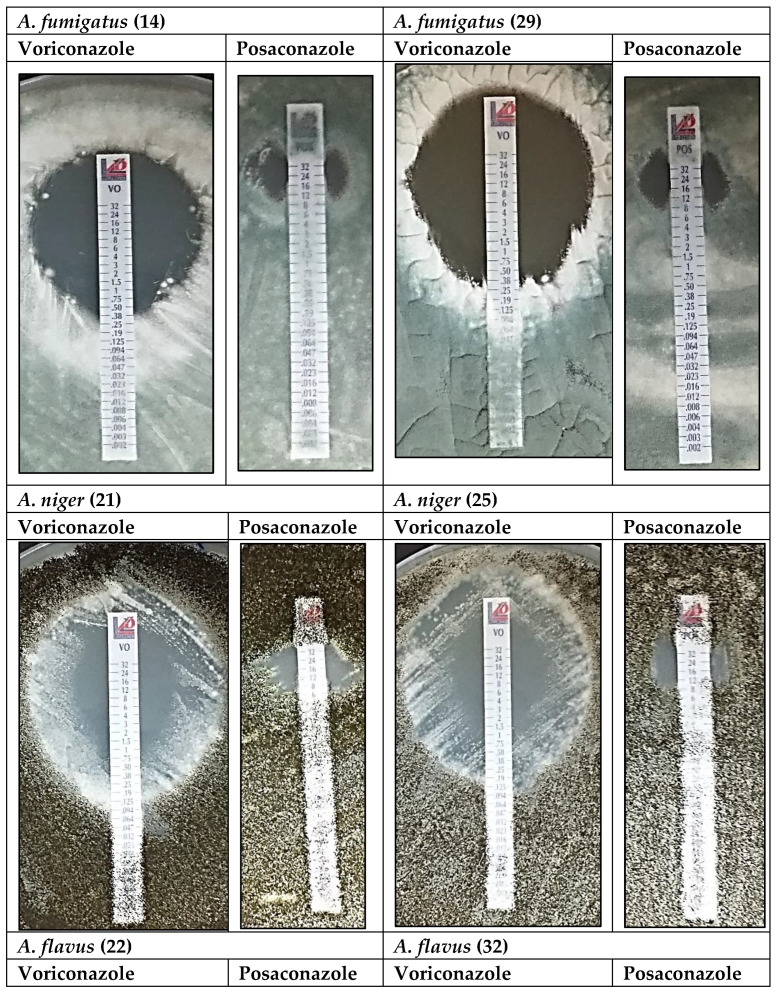

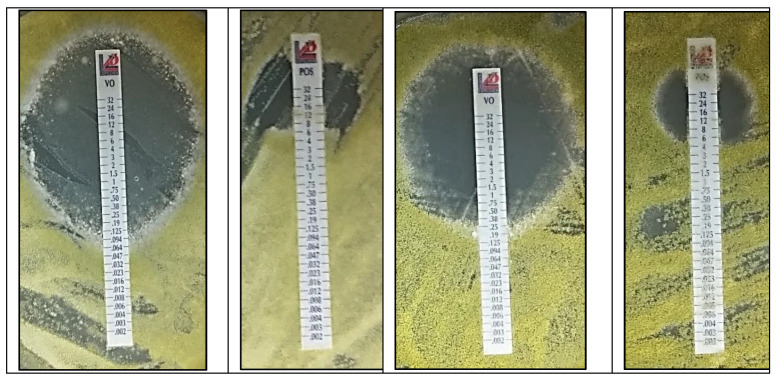
Effect of voriconazole and posaconazole on *A. fumigatus*, *A. niger* and *A. flavus* at 48 h. MIC Test Strip with azole concentration from 0.002–32 μg/mL.

Minimum inhibitory concentration (MIC) is important to understand the susceptibility of organisms or the effectiveness of chemicals used for infection treatment. In this study, azole-impregnated strips were used to assess the susceptibility/resistance of the clinical isolates to known azole therapeutics. The MIC and inhibition zones of voriconazole and posaconazole on all *Aspergillus* species were recorded as resistance (>2.0 μg/mL), susceptibility (≤0.25 μg/mL) and intermediate susceptibility (>0.25–2.0 μg/mL) for voriconazole. For posaconazole, isolates were recorded as resistant if they were to grow above 0.25 μg/mL and susceptibility if ≤0.06 μg/mL and intermediate susceptibility from >0.06–0.25 μg/mL. The results are reported in Table 2 for *A. fumigatus*, *A. niger*, and *A. flavus*. Two isolates of each species were selected to represent typical reactions on the MIC of voriconazole and posaconazole at 48 h of incubation (Figure 3).

MICs for isolates 14 and 29 for example (*A. fumigatus*), were 0.25 μg/mL for voriconazole (susceptible ≤ 0.25) and 12.0 μg/mL and 8.0 μg/mL (resistant > 0.25), respectively, for posaconazole as can be seen in Table 2. This was the overall trend observed in *A. fumigatus* strains, with susceptibility to voriconazole and high resistance well past the minimum concentration of 0.25 μg/mL to posaconazole, raising concern over this emerging resistance to salvage therapy, posaconazole.

*Aspergillus niger* isolates 21 and 25 showed moderate susceptibility (1.0 μg/mL) to voriconazole. Voriconazole was more effective on *Aspergillus niger* than posaconazole, with a MIC of 1.0 μg/mL for voriconazole and 8.0 μg/mL for posaconazole (Figure 3). Higher voriconazole concentrations resulted in much larger inhibition zones (34 mm and 39 mm) than MIC inhibition zones. This was not the same for posaconazole. For isolate 18, 12 mm was achieved with 16 μg/mL and 14 mm with 24 μg/mL (Table 2).

Again, voriconazole was more effective on *Aspergillus flavus* than posaconazole for isolates 22 and 32 (Figure 3). MICs (0.5 and 1.5 μg/mL) were higher than for *A. fumigatus* but much lower than for *A. niger*. Like *A. niger*, higher voriconazole concentrations (18 and 12 μg/mL) resulted in much larger inhibition zones (39 and 31 mm) but not for posaconazole. A concentration of 24 μg/mL resulted in only 21 and 18 mm inhibition zones for *A. flavus* (Table 2).

The results of voriconazole and posaconazole on *A. fumigatus* reveal 11 isolates susceptible (≤0.25 µm) to voriconazole (green) and 15 isolates with intermediate susceptibility (>0.25–2.0 µm), highlighted in yellow. All 26 isolates were resistant (>2.0 µm) to posaconazole(blue), and recorded resistance between 1.0 µg/mL to 16.0 µg/mL. The most significant inhibition zones of 34 mm for *A. fumigatus*, resulted from 16 ug/mL concentration, as seen with isolates 4, 5, and 34, shaded grey. The 26 *A. fumigatus* isolates recorded the lowest effective concentration from 0.19 µg/mL to 1.5 µg/mL for voriconazole resulting in 42% susceptible isolates and 58% with intermediate susceptibility (Table 2).

Voriconazole on *A. niger* showed 12.5% susceptibility (≤0.25 µm), 62.5% intermediate susceptibility (>0.25–2.0 µm), and 25% resistance (>2.0 µm) to voriconazole. Of the eight *A. niger* isolates, one was sensitive (green), five showed intermediate sensitivity (yellow), and two were resistant (blue) to voriconazole. Isolates 12 and 30 are resistant strains. All isolates were resistant to posaconazole recording resistance at concentrations of 8.0 μg/mL and 12.0 μg/mL. A*. niger* isolates revealed larger inhibition zones at higher concentrations of the azole. Isolate 21 and 33 had over 40 mm inhibition at 8 μg/mL (shaded). These results, in vitro, show voriconazole was effective on *A. niger* isolates at higher concentrations (Table 2). Both isolates of *A. flavus* showed intermediate susceptibility (>0.25–2.0 µg) to voriconazole and were resistant to posaconazole (>2.0 µg), as seen in Table 2. Both *A. flavus* strains showed intermediate susceptibility (yellow) to voriconazole but one-third of the concentration (0.5 µg) was effective on isolate 22 when compared to isolate 32 (1.5 µg).

### 3.3. Phenotypic Microarray of Antifungal Compounds

Twenty-four compounds (four wells each) made up the 96-well panel of antifungal compounds tested on the isolates. Growth data was captured and recorded using the Biolog Microlog system at 24-hr intervals over a 96 h period at 750 nm. However, 48 h readings were selected for analysis for isolates to be compared for their chemical response. Analysis of the response of the 36 *Aspergillus* isolates in the presence of 24 potential antifungal agents was visualized using a heatmap (Figure S3) presented in the Supplementary file.

**Figure 4:**
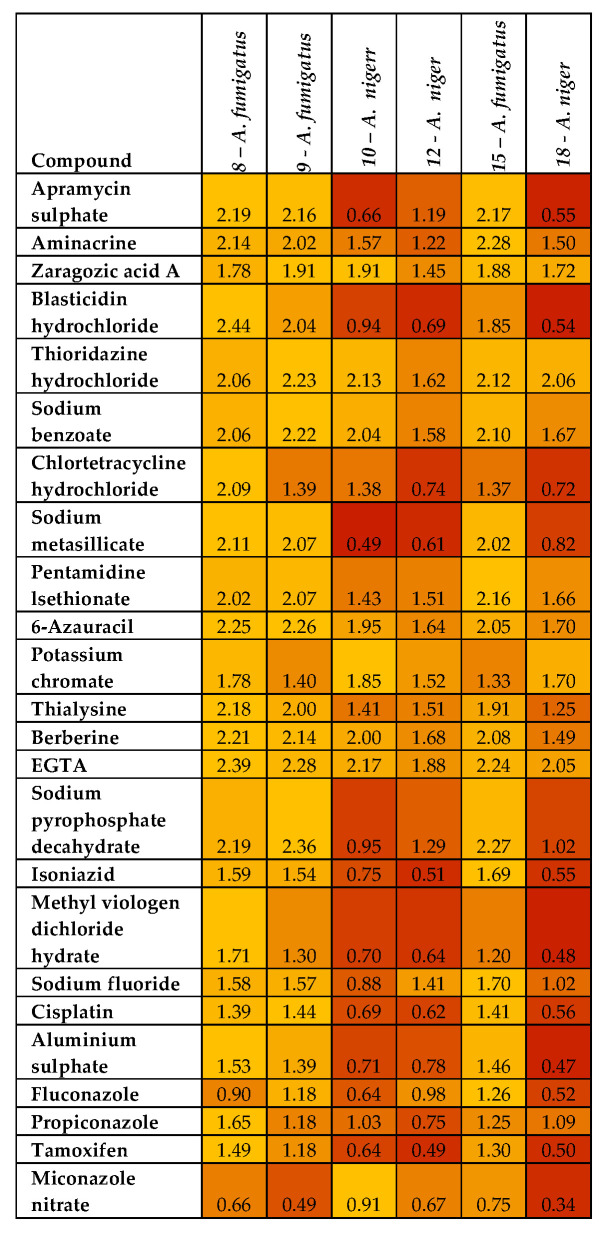
Absorbance readings taken at 750nm after 48hr of incubation of isolates (8, 9, 10,12, 15, 18) in a grown medium incorporated with the 24 compounds (Biolog). Red indicates susceptibility of isolates and yellow the resistance of isolates to the potential antifungal compounds.

Resistance to most compounds by *A. fumigatus* isolates (8, 9, and 15) and *A. niger* isolates (10, 12, and 18) could be observed in Figure 4. The three *A. fumigatus* isolates were resistant to all 24 compounds and the three *A. niger* isolates were resistant to more than 50% of the 24 compounds. The resistant *A. fumigatus* (isolate 8) had robust growth absorbance of 2.475 in 6-Azauracil. The remaining thirty isolates were generally susceptible to all 24 compounds (Figure S3—Supplementary file). Of these susceptible isolates, 63% of the *A. niger* strains (*n* = 8), 88% of the *A. fumigatus* strains (*n* = 26), and the *A. flavus* strains had varying susceptibility to the 24 compounds with absorbance generally below 0.50, showing a significant inhibitory action and potential for antifungal therapy.

### 3.4. Identification of TR34/98 Mutations Associated with Azole Resistance

Identification of TR34/98 mutations of the *cyp51A* gene, a major marker of azole genetic resistance, was conducted. The substitution of leucine to histidine mutation at position 98 in the protein sequence is usually associated with *A. fumigatus* strains becoming resistant to azole therapy [28]. The amino acid alignment revealed that 42% (11 strains) contained the TR34/98 mutation, while the rest (58%, 15 strains) contained the wild-type *cyp51A* gene (Table 3). However, although 15 isolates had wild-type genes, only 31% were susceptible to voriconazole and 27% with moderate resistance. Of the eleven mutated *A. fumigatus* isolates, 73% showed intermediate susceptibility (≤0.25 μm) to voriconazole and three strains (27%) remained susceptible even though the strains recorded mutations. The wild-type *A. fumigatus* strains showed intermediate susceptibility (>0.25–2.0 μm) but at higher concentrations between 0.50 and 1.50 μm for six of the isolates (8, 15, 23, 26, 27, and 35) (Table 3). The wild-type and mutated strains of all three species showed resistance to posaconazole. To ascertain a trend of resistance in the isolates due to mutations would require a larger sample size which is not favored with this number of isolates in conjunction with the analysis of other markers for resistance.

## 4. Discussion

The editorial in National Microbiology (2017) highlighted the issue of fungal infection neglect, estimating the global prevalence of life-threatening fungal infections to be over 300 million per year, resulting in 1.6 million fatalities annually [29]. Alarmingly, severe fungal infections are on the rise, and new drugs to treat them are coming to market at an unacceptably slow rate. As a result, to decrease or stabilize infection management from detection and diagnosis through therapy, should be addressed. The rapid initiation of effective antifungal therapy dramatically decreases mortality in invasive aspergillosis, making identification and characterization of drug resistance in vitro critical in treatment [30]. Clinical success in managing azole resistance is determined by advancements in all aspects, from detection and antifungal therapy to the discovery of novel, non-toxic medicines. As a result, the purpose of this study was to analyze the effect of regular and salvage drug therapy on *Aspergillus* species related to respiratory infections, as well as compare conventional to DNA-based identification methods and the novel phenotypic Biolog techniques for rapid identification. Phenotypic microarray was also employed for novel antifungal drug screening, substituting the labor-intensive biochemical assays used in normal analysis to assess the *Aspergillus* species’ carbon nutrition profile and better understand resistance patterns.

This study evaluated the conventional methods of culture and microscopic examination of morphological features, 18S rRNA, and a high throughput phenotypic/biochemical identification system in identifying clinical isolates to address this aspect of identification and diagnosis in the management of IA. Although DNA-based technologies are becoming more accessible, microscopy and culture techniques remain the principal laboratory tools for first identification. DNA-based identification has gained reliability and confidence and is a “gold standard” for microbial identification [31]. However, cost and affordability are factors, and thus morphological methods still serve a purpose and are of value in many settings, as demonstrated in this study, where phenotypic identification matched identification by 18S rRNA, although when fungal species occurred as cryptic species, morphological method was unable to resolve this. Cryptic species are species that are difficult to distinguish or identify based on morphology alone [32]. The ability to identify such culture is crucial when choosing therapeutic chemical agents for disease control [33]. Of the 36 isolates, 26 were identified as *A. fumigatus*, eight *A. niger*, and two *A. flavus* using culture, and microscopy and DNA identification were in agreement except for isolates 12, 13, and 21 which contained cryptic species.

In this investigation, conventional identification was useful; nevertheless, the number of isolates was small compared to the vast diversity of fungal species capable of causing human diseases. Isolating fungi that grow close together is a complex and time-consuming process when working with large numbers and contributes to diagnostic delays. This is a concern in developing countries where the disease burden is much higher. The solution to this problem is to improve diagnostic platforms so that infecting fungi can be identified faster and with greater accuracy and precision. However, in the search for new diagnostic technologies, training in mycology for fungal identification based on morphologic and phenotypic characteristics is further diminished. When looking for accurate and fast alternatives in fungal identification, especially when detecting human pathogens affecting diagnosis, the phenotypic microarray for fungal species identification is a suitable option. The Biolog system used in this study is a relatively new technology that provides valuable extra results with a large quantity of support data. This supporting data are a benefit of the Biolog system over molecular identification, which has gained confidence in the dependability of the results but provides no supporting data. However, the Biolog system’s species identification did not match the species level identification established using 18S rRNA, which corresponded with conventional culture and microscopy identification in this investigation. Although the Biolog database contains clinically related *Aspergillus* species, the database most likely did not contain closely correlated clinical species with the same biochemical profile at the time of usage, resulting in low and insignificant similarity readings. Most isolates were matched, but only at the genus level, which is ineffective in clinical settings (Table 1). This system’s restriction or disadvantage for recognizing isolates is only as good as its reference database for effective utilization. This technique and procedure for species identification are relatively simple and quick, and they would have been useful if the database included more clinically important *Aspergillus* species. However, with its extended database, the Biolog system may give an alternative for detecting filamentous fungus where molecular approaches are ineffective.

Each identification technique investigated in this study could be used in different conditions depending on the financial support and skills available. Classical mycological procedures based on phenotypic traits should continue to develop expertise to enable correct identification where molecular techniques and other modern equipment-based techniques are not available. However, molecular techniques, although reliable and consistent, require specialized and advanced equipment and software and are, therefore, sometimes not accessible to the developing world. Cost becomes a factor in considering isolates could be sent externally for sequencing. The Biolog system is a simple and quick identification technique that relies on a database; nevertheless, the system must be able to incorporate new species into the diagnostic capabilities and should be continuously updated.

Despite the difficulties in detecting *Aspergillus* in respiratory infections, the most concerning factor remains azole resistance in *Aspergillus*. These life-threatening diseases strike the most vulnerable patient populations. These fungi have emerged as a major cause in people who have underlying medical conditions or are undergoing immunosuppressive therapy [13]. The increased prevalence of invasive fungal infections has coincided with a significant increase in azole resistance in *Aspergillus* [12] and fungal infections are spiraling, with an estimated 1.5 million human mortalities yearly [34,35]. Effective antifungal therapy is critical in treating the disease in patients with compromised immune systems, particularly in cases of invasive pulmonary aspergillosis, which can progress quickly. As a result, this study evaluated the efficacy of voriconazole and posaconazole, which are presently employed in the normal treatment of *Aspergillus* infections in respiratory patients, as well as the potential of new antifungal agents in azole resistance. Voriconazole and posaconazole are broad-spectrum triazole antifungal medicines used to treat invasive fungal infections [23]. Voriconazole is used for routine therapy and is the first-line agent for the treatment of IA [36] and posaconazole as salvage therapy for *Aspergillus* infections [37].

This investigation shows that 6% of the *Aspergillus* isolates were resistant to the standard drug voriconazole, whereas 61% exhibited moderate sensitivity. Alarmingly, all isolates were resistant to the salvage therapy drug posaconazole (Table 2). These findings are concerning because azoles have previously improved the treatment of IA and reduced mortality rates [37]. As a result of growing resistance, further techniques for controlling mortality and morbidity must be studied. Authors of [38] discovered triazole resistance in 16% of patients with influenza-related IA, and voriconazole resistance was substantially linked with treatment failure. Although not many *A. fumigatus* isolates were resistant to voriconazole in this investigation, more than half (58%) exhibited moderate sensitivity (>0.25–2.0 μg/mL). Thus, *Aspergillus* susceptibility to voriconazole reported in the above investigations, as a first-line therapy azole, requires vigilance for triazole-resistant strains, especially since *A. fumigatus* is responsible for over 90% of cases of invasive aspergillosis [37,39].

Twenty-five per cent of the *A. niger* isolates are resistant to voriconazole and show more resistance to voriconazole than *A. niger* and *A. flavus*. This is especially significant because the only isolates resistant to voriconazole and posaconazole in this study are two *A. niger* strains (isolates 12 and 30) (Table 2). It is well-documented and reported that *A. fumigatus* is the most commonly isolated species from respiratory infections [37], with azole resistance recorded as 1.5% in the United States [40] and resistance extending beyond Europe to other continents [41]. However, it has to be noted that *A. niger* made up 14.3% of *Aspergillus* species recovered from pulmonary aspergillosis infections in patients (*n* = 71) with HIV in Northern India [42] and in this current study, 22% of the isolates from respiratory infections (*n* = 36), collected between July 2015 and June 2016. It is thus significant that *A. niger* is emerging in respiratory infections and recording high resistance, as recorded here with 25% resistance to voriconazole and 100% resistance to posaconazole. Although *A. fumigatus* is the most commonly isolated species from respiratory infections and increasing azole resistance [43], *A. niger* may pose a more significant challenge in the not-so-distant future if not monitored and effective measures are not put in place. The resistance of all isolates to posaconazole is a serious concern as the survival rate of immune-compromised patients with invasive aspergillosis depends on the availability of multiple options for antifungal drugs [44]. Thus, the resistance observed with posaconazole threatens the overall effectiveness of the therapy strategy.

*Aspergillus* resistance associated with adapting environmental isolates to human pathogens must be considered in the emergence of this resistance due to the widespread use of agricultural azoles [45]. Azoles are commonly used for crop protection and some fungicides have a molecular structure similar to that of medical triazoles [46]. Thus it has been suggested that mutations that confer resistance to fungicides can offer resistance to medical azoles due to the molecule’s structural similarity [47]. Plant-pathogenic molds that cause opportunistic infections in humans are exposed to demethylase inhibitor (DMI) fungicides, including imidazole and triazole drugs. As a result, a loss of efficacy has occurred for this drug class in several species. With the global increase in the use of DMI fungicides, a rise in the number of *A. fumigatus* azole-resistant isolates has been reported [48]. The azole drugs act by inhibiting the activity of *cyp51* enzymes, the azole target. Mutations in the *cpy51A* gene represent common mechanisms for azole resistance in clinical *Aspergillus* isolates [49]. In this study, of the eleven *A. fumigatus* isolates that were susceptible (≤0.25 μm) to voriconazole, eight isolates (73%) were the wild type and only three of those susceptible strains remained susceptible even though the strains recorded mutations (Table 3). The other fifteen *A. fumigatus* strains showed moderate susceptibility (>0.25–2.0 μm) and at higher concentrations between 0.50 and 1.50 μm, for six of the isolates. Two of those isolates, 15 and 27 are mutated strains and could be developing resistance due to the mutation. Therefore, it seems that maybe different genetic mutations offer resistance to azole than the tr34/96 tested here. Mutations at positions G54, M220, and G448 of the *cyp51A* gene have been reported to be mutation hot spots and were observed in patients with chronic aspergillosis treated with azole antifungals in the long term [20,50]. Other less common point mutations (G138C and F219I) were found in patients receiving long-term treatment with azole antifungals [20,51].

In light of emerging antifungal resistance, in an attempt to increase the therapeutic range, effective alternative compounds were explored to improve therapeutic options such as combination therapy, as suggested by [52,53], to reduce the rate of resistance and increase potency. This antifungal phenotypic study was achieved by analyzing the sensitivity profile of the isolates in a microarray system (Biolog). The profile results show the resistant compounds in yellow and susceptibility in red on the heat map (Figure 4). Exposure of the *Aspergillus* isolates to the potential antifungal agents showed that 83% of the isolates were susceptible to the 24 compounds. The large number of isolates being susceptible to the 24 compounds and although in varying degrees of susceptibility, shows promise in the quest to find new therapies for fungal resistance that could impact mortality rates that are as high as 90% in invasive fungal infections due to limited treatment [53]. However, the agents must be considered for toxicity for oral consumption. In this study, two compounds, berberine and blasticidin hydrochloride, from the agents assessed in the current study were effective against thirty of the isolates. This is promising because this could offer an alternative, or azoles could be assessed for combination therapy with either or both of these agents as a potential option, seeing that azole monotherapy has led to resistance after initial success [54]. Berberine and blasticidin hydrochloride from the compounds assessed were also part of the [53] investigation with fluconazole, resulting in an antagonistic and synergistic effect, respectively. Berberine is a plant extract that reduces blood sugar levels [55,56]. Both compounds have low toxicity at therapeutic concentrations and may be suitable candidates for trials in combination therapy, with the escalating concern of azoles showing resistance in monotherapy. Blasticidin hydrochloride is a gene selection antibiotic effective in eucaryotic cells, inhibiting the peptide bond of ribosomes [57]. Increasing the therapeutic range and exploring effective alternative compounds in combination therapy is important and could reduce the resistance rate and increase potency [51,52].

## 5. Conclusions

This study showed that phenotypic microarray is a potentially rapid and reliable tool for fungal identification in impacting effective diagnosis in azole resistance management. This investigation has also highlighted drug resistance in azole monotherapy after initial success and thus explored the potential of new agents using phenotypic microarray in drug regimen strategy for combination therapy.

## Figures and Tables

**Table 1:** Results of the identification of the isolates to species level by microscope and culture techniques and 18S rRNA (column 2). The Biolog Identification System results are in column 3 with the low percentage similarity match at the species level by the Biolog Identification System in the fourth column. The background color indicates the clusters within which the different isolates belong on the phylogenic tree (Figure 2).

Isolate	18S rRNA/Microscope and Culture Techniques	Biolog Identification (48 h Incubation)	Similarity 1.0 = 100%
1	*Aspergillus fumigatus*	*Neosartorya ficheri*, *Petromyces alliaceus*, *Aspergillus versicolor*, *Aspergillus clavatus*, *Aspergillus tamarii*, *Aspergillus parasiticus*,	<0.1
2	*Aspergillus fumigatus*	*Neosartorya ficheri*, *Petromyces alliaceus*, *Aspergillus parasiticus*, *Aspergillus tamarii.*	<0.1
3	*Aspergillus fumigatus*	*Neosartorya ficheri*, *Aspergillus tamarii*, *Aspergillus versicolor*, *Aspergillus puniceus*, *Petromyces alliaceus*	<0.1
4	*Aspergillus fumigatus*	*Neosartorya ficheri*, *Petromyces alliaceus Aspergillus tamarii*, *Aspergillus versicolor*, *Aspergillus parasiticus*	<0.1
5	*Aspergillus fumigatus*	*Neosartorya ficheri*, *Petromyces alliaceus Aspergillus tamarii*, *Aspergillus versicolor*, *Aspergillus parasiticus*	<0.1
6	*Aspergillus fumigatus*	*Neosartorya ficheri*, *Petromyces alliaceus*, *Aspergillus puniceus*, *Aspergillus tamarii*, *Aspergillus parasiticus*	<0.1
7	*Aspergillus fumigatus*	*Neosartorya ficheri*, *Petromyces alliaceus*, *Aspergillus tamarii*, *Aspergillus parasiticus*, *Aspergillus puniceus*	<0.1
8	*Aspergillus fumigatus*	*Neosartorya ficheri*, *Petromyces alliaceus*, *Aspergillus tamarii*, *Aspergillus aureolatus*, *Aspergillus versicolor*	<0.1
9	*Aspergillus fumigatus*	*Aspergillus parasiticus*, *Petromyces alliaceus*, *Neosartorya ficheri*, *Aspergillus tamarii*, *Chaetosartorya stomatoides.*	<0.1
10	*Aspergillus niger*	*Aspergillus niger*, *Chaetosartorya stomatoides*, *Aspergillus ustus*, * Emericella variecola*, *Neosartorya ficheri*	<0.1 *A. niger* = 0.006
11	*Aspergillus fumigatus*	*Petromyces alliaceus*, *Neosartorya ficheri*, *Aspergillus tamarii*, *Aspergillus parasiticus*	<0.1
12	*Aspergillus niger/tubingensis*	*Aspergillus niger*, *Neosartorya ficheri*,*Aspergillus parasiticus*, *Aspergillus ustus*, *Chaetosartorya stomatoides*, *Aspergillus foetidus*, *Emericella variecola*	<0.1 *A. niger* = 0.086 *C. stromatoides* = 0.138
13	*Aspergillus niger/tubingensis*	*Emericella striata*, *Emericella fruticulosa*, *Aspergillus wentii*, *Aspergillus phoenicis*, *Aspergillus brasiliensis*, *Emericella violacea*, *Aspergillus zonatus*	<0.1
14	*Aspergillus fumigatus*	*Petromyces alliaceus*, *Neosartorya ficheri*, *Aspergillus tamarii*, *Aspergillus parasiticus*, *Aspergillus terricola*, *Aspergillus flavus*	<0.1 *A. terricola* < 0.349
15	*Aspergillus fumigatus*	*Aspergillus terricola*, *Aspergillus flavus*, *Aspergillus pulverulentus*, *Chaetosartorya stomatoides*, *Aspergillus sydowii*	<0.1 *A. terricola* = 0.565
16	*Aspergillus fumigatus*	*Aspergillus terricola*, *Aspergillus flavus*, *Aspergillus pulverulentus*, *Neosartorya ficheri*, *Aspergillus sydowii*	<0.1 *A. terricola* = 0.371
17	*Aspergillus fumigatus*	*Aspergillus terricola*, *Aspergillus flavus*, *Neosartorya ficheri*, *Aspergillus sydowii*	<0.1 *A. terricola* = 0.514
18	*Aspergillus niger*	*Aspergillus brasiliensis*, *Aspergillus kanagawaensis*, *Emericella striata*, *Aspergillus restrictus*, *Aspergillus zonatus*, *Emericella violacea*	<0.1
19	*Aspergillus fumigatus*	*Aspergillus terricola*, *Aspergillus flavus*, *Neosartorya ficheri*, *Aspergillus versicolor*, *Chaetosartorya stomatoides*	<0.1 *A. terricola* = 0.137
20	*Aspergillus fumigatus*	*Aspergillus puniceus*, *Chaetosartorya stomatoides*, *Aspergillus ustus*, *Aspergillus terricola*, *Aspergillus flavus*, *Neosartorya ficheri*	<0.1 *A. puniceus* = 0.167
21	*Aspergillus niger/welwitschiae*	*Aspergillus foetidus*, *Emericella violacea*, *Aspergillus niger*, *Aspergillus brasiliensis*, *Emericella striata*, *Aspergillus ochraceus*, *Aspergillus carneus*	<0.1 *A. foetidus* = 0.130 *A. niger* = 0.003
22	*Aspergillus flavus*	*Aspergillus terricola*, *Aspergillus flavus*, *Neosartorya ficheri*, *Aspergillus pulverulentus*,	<0.1 *A. terricola* = 0.148 *A. flavus* = 0.002
23	*Aspergillus fumigatus*	*Aspergillus terricola*, *Aspergillus flavus*, *Chaetosartorya stomatoides*, *Aspergillus puniceus*, *Neosartorya ficheri*, *Aspergillus versicolor*	<0.1 *A. terricola* =0.407
24	*Aspergillus fumigatus*	*Aspergillus terricola*, *Aspergillus flavus*, *Neosartorya ficheri*, *Aspergillus puniceus*, *Chaetosartorya stomatoides*,	<0.1 *N. ficheri* = 0.186
25	*Aspergillus niger*	*Aspergillus foetidus*, *Aspergillus niger*, *Emericella violacea*, *Aspergillus wenti*, *Aaspergillus brevipes*, *Aspergillus carbonarius*, *Aspergillus brasiliensis*, *Chaetosartorya stomatoides*	<0.1 *A. foetidus* = 0.116 *A. brevipes* = 0.145 *A. niger* = 0.015
26	*Aspergillus fumigatus*	*Aspergillus flavus*, *Neosartorya ficheri*, *Aspergillus terricola*, *Chaetosartorya stomatoides*, *Neosartorya ficheri*	<0.1 *A. flavus* = 0.350 *A. terricola* = 0.205
27	*Aspergillus fumigatus*	*Aspergillus terricola*, *Aspergillus flavus*, *Aspergillus puniceus*, *Aspergillus versicolor*, *Neosartorya ficheri*, *Aspergillus pulverulentus*	<0.1 *A. terricola* = 0.240
28	*Aspergillus fumigatus*	*Aspergillus terricola*, *Aspergillus flavus*, *Aspergillus versicolor*, *Aspergillus pulverulentus*, *Neosartorya ficheri*	<0.1 *A. terricola* =0.332
29	*Aspergillus fumigatus*	*Aspergillus terricola*, *Aspergillus flavus*, *Aspergillus versicolor*, *Neosartorya ficheri*, *Aspergillus pulverulentus*	<0.1 *A. terricola* = 0.238
30	*Aspergillus niger*	*Aspergillus niger*, *Emericella violacea*, *Aspergillus carbonarius*, *Aspergillus foetidus*, *Aspergillus brasiliensis*, *Emericella striata*, *Aspergillus zonatus*, *Aspergillus carbonarius*	<0.1 *A. niger* = 0.008
31	*Aspergillus fumigatus*	*Aspergillus terricola*, *Aspergillus puniceus*, *Chaetosartorya stomatoides*, *Aspergillus flavus*, *Aspergillus ustus*	<0.1 *A. terricola* =0.296
32	*Aspergillus flavus*	*Aspergillus terricola*, *Aspergillus flavus*, *Neosartorya ficheri*, *Aspergillus pulverulentus*, *Aspergillus puniceus*,	<0.1 *A. terricola* = 0.347 *A. flavus* = 0.000
33	*Aspergillus niger*	*Aspergillus ustus*, *Aspergillus versicolor*, *Emericella violacea*, *Aspergillus sydowii*, *Aspergillus caesipitosus*, *Aspergillus foetidus*,	<0.1 *A. caespitosus* = 0.187
34	*Aspergillus fumigatus*	*Aspergillus terricola*, *Aspergillus flavus*, *Aspergillus puniceus*, *Neosartorya ficheri*	<0.1 *A. flavus* = 0.162 *A. terricola* =0.152
35	*Aspergillus fumigatus*	*Aspergillus terricola*, *Aspergillus flavus*, *Aspergillus pulverulentus*, *Chaetosartorya stomatoides*	<0.1 *A. terricola* = 0.472
36	*Aspergillus fumigatus*	*Aspergillus puniceus*, *Neosartorya ficheri*, *Aspergillus ustus*, *Aspergillus flavus*, *Chaetosartorya stomatoides*, *Aspergillus fumigatus*	<0.1 *A. puniceus* = 0.227 *N. ficheri* = 0.247 *A. fumigatus* = 0.000

**Table 2:** The lowest effective concentration (MIC in µg/mL) of voriconazole and posaconazole *A fumigatus*, *A. niger* and *A. flavus* and the concentration (µg/mL) of voriconazole and posaconazole that produced the largest inhibition of growth in mm after 48 h of incubation at 37 °C. Voriconazole: MIC ≤ 0.25; intermediate susceptibility > 0.25–2.0; resistant > 2.0. Posaconazole: MIC ≤ 0.06; intermediate susceptibility > 0.06–0.25; resistant > 0.25.

		Voriconazole		Posaconazole	
Isolate No.	Species	Lowest Effective Concentration (µg/mL)	Concentration (µg/mL) for the Largest Inhibition Zone (mm)	Lowest Effective Concentration (µg/mL)	Concentration (µg/mL) for the Largest Inhibition Zone (mm)
1	*A. fumigatus*	0.19	12.0–29	16.0	24.0–10
2	*A. fumigatus*	0.38	12.0–30	16.0	24.0–10
3	*A. fumigatus*	0.38	12.0–31	12.0	24.0–11
4	*A. fumigatus*	0.25	16.0–34	12.0	24.0–12
5	*A. fumigatus*	0.19	16.0–34	12.0	24.0–12
6	*A. fumigatus*	0.38	12.0–28	12.0	24-0–13
7	*A. fumigatus*	0.38	12.0–27	12.0	24.0–13
8	*A. fumigatus*	0.50	12.0–28	16.0	24.0–12
9	*A. fumigatus*	0.38	12.0–29	12.0	24.0–12
11	*A. fumigatus*	0.38	12.0–31	12.0	24.0–17
14	*A. fumigatus*	0.25	12.0–29	12.0	24.0–15
15	*A. fumigatus*	0.50	12.0–28	8.0	16.0–13
16	*A. fumigatus*	0.38	12.0–29	8.0	24.0–17
17	*A. fumigatus*	0.19	12.0–32	12.0	24.0–12
19	*A. fumigatus*	0.38	12.0–30	6.0	16.0–15
20	*A. fumigatus*	0.25	12.0–29	8.0	24.0–17
23	*A. fumigatus*	0.50	12.0–38	8.0	16.0–16
24	*A. fumigatus*	0.25	8..0–29	8.0	16.0–16
26	*A. fumigatus*	0.50	23.0–28	6.0	16.0–17
27	*A. fumigatus*	0.75	12.0–29	8.0	24.0–16
28	*A. fumigatus*	0.25	12.0–30	8.0	16.0–12
29	*A. fumigatus*	0.25	12.0–32	7.5	12.0–24
31	*A. fumigatus*	0.25	12.0–32	8.0	24.0–19
34	*A. fumigatus*	0.38	16.0–34	6.0	16.0–17
35	*A. fumigatus*	1.5	16.0–31	8.0	24.0–15
36	*A. fumigatus*	0.19	12.0–30	1.0	16.0–21
10	*Aspergillus niger*	0.19	12.0–39	8.0	16.0–13
12	*Aspergillus niger/tubingensis*	2.0	16.0–16	12.0	32.0–16
13	*Aspergillus niger/tubingensis*	1.5	12.0–21	12.0	16.0–13
18	*Aspergillus niger*	1.0	12.0–34	8.0	16.0–12
21	*Aspergillus niger/welwitschiae*	1.0	8.0–40	8.0	16.0–14
25	*Aspergillus niger*	1.0	8.0–39	8.0	24.0–14
30	*Aspergillus niger*	2.0	12.0–39	8.0	24.0–17
33	*Aspergillus niger*	1.5	8.0–43	12.0	34.0–13
22	*Aspergillus flavus*	0.5	8.0–39	6.0	24.0–21
32	*Aspergillus flavus*	1.5	12.0–31	6.0	24.0–18

**Table 3:** Minimum inhibitory concentration of voriconazole (VRC) against *Aspergillus fumigatus* and posaconazole (POS) and mutations in the cyp51A gene. The isolates containing the L98H are highlighted in blue.

Isolate No.	Species	Resistant (R)/Susceptible (S)/ Intermediate Susceptibility (IS)		*cyp51A* Mutations
		VRC	POS	
1	*A. fumigatus*	S	R	WT
2	*A. fumigatus*	IS	R	L98H
3	*A. fumigatus*	IS	R	L98H
4	*A. fumigatus*	S	R	L98H
5	*A. fumigatus*	S	R	WT
6	*A. fumigatus*	IS	R	L98H
7	*A. fumigatus*	IS	R	L98H
8	*A. fumigatus*	IS	R	WT
9	*A. fumigatus*	IS	R	L98H
10	*A. niger*	S	R	ND
11	*A. fumigatus*	IS	R	WT
12	*A. niger*	R	R	ND
13	*A. niger*	IS	R	ND
14	*A. fumigatus*	S	R	WT
15	*A. fumigatus*	IS	R	L98H
16	*A. fumigatus*	IS	R	WT
17	*A. fumigatus*	S	R	WT
18	*A. niger*	IS	R	ND
19	*A. fumigatus*	IS	R	WT
20	*A. fumigatus*	S	R	L98H
21	*A. niger*	IS	R	ND
22	*A. flavus*	IS	R	ND
23	*A. fumigatus*	IS	R	WT
24	*A. fumigatus*	S	R	L98H
25	*A. niger*	IS	R	ND
26	*A. fumigatus*	IS	R	WT
27	*A. fumigatus*	IS	R	L98H
28	*A. fumigatus*	S	R	WT
29	*A. fumigatus*	S	R	WT
30	*A. niger*	R	R	ND
31	*A. fumigatus*	S	R	WT
32	*A. flavus*	IS	R	ND
33	*A. fumigatus*	IS	R	WT
34	*A. fumigatus*	IS	R	L98H
35	*A. fumigatus*	IS	R	WT
36	*A. fumigatus*	S	R	WT

WT: wild-type. ND: not determined.

## Data Availability

The sequencing data will be made available when the manuscript is publicly available.

## Supplementary Materials

The following supporting information can be downloaded at: https://www.mdpi.com/article/10.3390/jof9060616/s1. Figure S1: Carbon sources in FF microplate with 95 wells, containing water as a control. Figure S2: Liofilchem MIC test strip with 0.002–32 μg/mL posaconazole. Figure S3: Absorbance readings taken at 750 nm after 48 h of incubation of isolates in a growth medium incorporated with the 24 compounds. Red indicates susceptibility and yellow indicates the resistance of isolates to the potential antifungal compounds. Figure S4: Phylogenic tree showing isolates of *Aspergillus* species. The sequences were aligned and the tree was prepared using Neighbor-Joining analysis and visualized on Treebase. Dark lines represent the branches with more than 75% support with UPGMA compared to the NJ tree (1000 bootstrap).
