# Supplementary material for: Phenotypic Array for Identification and Screening of Antifungals against Aspergillus Isolates from Respiratory Infections in KwaZulu Natal, South Africa
Source: J Fungi (Basel). 2023 May 26;9(6):616. doi: 10.3390/jof9060616 (PMC10303164; doi:10.3390/jof9060616)
Supplement: Supplementary file 1 [file jof-09-00616-s001.zip › jof-2313228-supplementary.pdf]

A1<br>Water	A2<br>Tween 80	A3<br>N-Acetyl-D-Galactosamine	A4<br>N-Acetyl-D-Glucosamine	A5<br>N-Acetyl-D-Mannosamine	A6<br>Adonitol	A7<br>Amygdalin	A8<br>D-Arabinose	A9<br>L-Arabinose	A10<br>D-Arabitol	A11<br>Arbutin	A12<br>D-Cellobiose
B1<br>[illegible]-Cyclodextrin	B2<br>[illegible]-Cyclodextrin	B3<br>Dextrin	B4<br>i-Erythritol	B5<br>D-Fructose	B6<br>L-Fucose	B7<br>D-Galactose	B8<br>D-Galacturonic Acid	B9<br>Gentiobiose	B10<br>D-Gluconic Acid	B11<br>D-Glucosamine	B12<br>[illegible]-D-Glucose
C1<br>Glucose-1-Phosphate	C2<br>Glucuronamide	C3<br>D-Glucuronic Acid	C4<br>Glycerol	C5<br>Glycogen	C6<br>m-Inositol	C7<br>2-Keto-D-Gluconic Acid	C8<br>[illegible]-D-Lactose	C9<br>Lactulose	C10<br>Maltitol	C11<br>Maltose	C12<br>Maltotriose
D1<br>D-Mannitol	D2<br>D-Mannose	D3<br>D-Melezitose	D4<br>D-Melibiose	D5<br>[illegible]-Methyl-D-Galactoside	D6<br>[illegible]-Methyl-D-Galactoside	D7<br>[illegible]-Methyl-D-Glucoside	D8<br>[illegible]-Methyl-D-Glucoside	D9<br>Palatinose	D10<br>D-Psicose	D11<br>D-Raffinose	D12<br>L-Rhamnose
E1<br>D-Ribose	E2<br>Salicin	E3<br>Sedoheptulosan	E4<br>D-Sorbitol	E5<br>L-Sorbose	E6<br>Stachyose	E7<br>Sucrose	E8<br>D-Tagatose	E9<br>D-Trehalose	E10<br>Turanose	E11<br>Xylitol	E12<br>D-Xylose
F1<br>[illegible]-Amino-butyric Acid	F2<br>Bromosuccinic Acid	F3<br>Fumaric Acid	F4<br>[illegible]-Hydroxy-butyric Acid	F5<br>[illegible]-Hydroxy-butyric Acid	F6<br>p-Hydroxyphenyl-acetic Acid	F7<br>[illegible]-Keto-glutaric Acid	F8<br>D-Lactic Acid Methyl Ester	F9<br>L-Lactic Acid	F10<br>D-Malic Acid	F11<br>L-Malic Acid	F12<br>Quinic Acid
G1<br>D-Saccharic Acid	G2<br>Sebacic Acid	G3<br>Succinamic Acid	G4<br>Succinic Acid	G5<br>Succinic Acid Mono-Methyl Ester	G6<br>N-Acetly-L- Glutamic Acid	G7<br>Alaninamide	G8<br>L-Alanine	G9<br>L-Alanyl-Glycine	G10<br>L-Asparagine	G11<br>L-Aspartic Acid	G12<br>L-Glutamic Acid
H1<br>Glycyl-L-Glutamic Acid	H2<br>L-Ornithine	H3<br>L-Phenylalanine	H4<br>L-Proline	H5<br>L-Pyroglutamic Acid	H6<br>L-Serine	H7<br>L-Threonine	H8<br>2-Amino Ethanol	H9<br>Putrescine	H10<br>Adenosine	H11<br>Uridine	H12<br>Adenosine-5'-Monophosphate

Figure S1. Carbon sources in FF microplate with 95 wells, containing water as a control

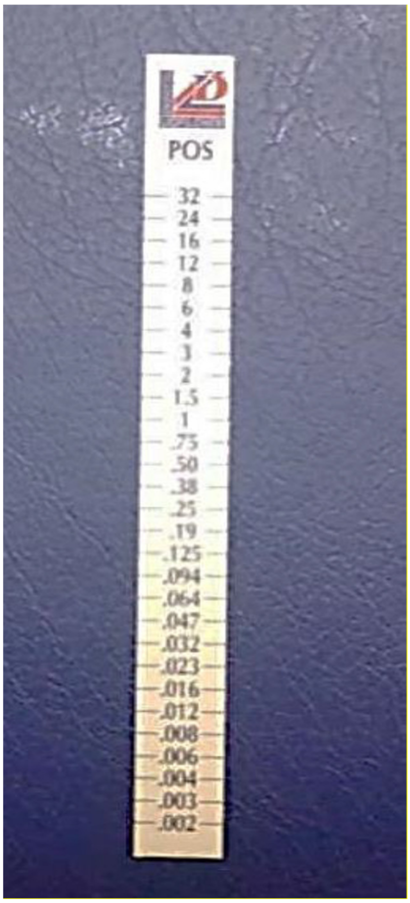

Figure S2. Liofilchem MIC test strip with 0.002-32 μg/ml posaconazole. The MIC test strip for voriconazole has the same concentration range

Compound	1 - A. fumigatus	2 - A. fumigatus	3 – A. fumigatus	4 – A. fumigatus	5 – A. fumigatus	6 – A. fumigatus	7 – A. fumigatus	8 – A. fumigatus	9 - A. fumigatus	10 – A.. nigerr	11 – A. fumigatus	12 - A . niger	13 - A . niger	14 – A. fumigatus	15 – A. fumigatus	16 – A. fumigatus	17 – A. fumigatus	18 - A.niger	19 – A. fumigatus	20 – A..fumigatus	21 - A . niger	22 - A. flavus	23 – A. fumigatus	24 – A. fumigatus	25 - A. . niger	26 - A. fumigatus	27 – A . fumigatus	28 - A. fumigatus	29 - A. fumigatus	30 - A.. niger	31 – A. fumigatus	32 - A. flavus	33 - A. niger	34 – A. fumigatus	35 – A. fumigatus	36 – A. fumigatus
Apramycin sulphate	0,315	0,278	0,253	0,4	0,201	0,249	0,281	2,198	2,16	0,662	0,232	1,198	0,451	0,253	2,175	0,685	0,324	0,555	0,33	0,226	0,233	0,326	0,269	0,286	0,265	0,233	0,24	0,361	0,706	0,19	0,32	0,274	0,225	0,35	0,35	0,36
Aminacrine	0,367	0,306	0,255	0,311	0,209	0,303	0,269	2,145	2,026	1,577	0,243	1,225	0,226	0,325	2,287	0,295	0,307	1,509	0,275	0,276	0,199	0,392	0,274	0,305	0,194	0,343	0,37	0,28	0,318	0,199	0,419	0,382	0,253	0,296	0,31	0,306
Zaragozic acid A	0,237	0,248	0,226	0,253	0,191	0,212	0,217	1,783	1,919	1,91	0,191	1,45	0,173	0,237	1,88	0,259	0,236	1,729	0,243	0,203	0,174	0,289	0,221	0,249	0,167	0,226	0,233	0,265	0,272	0,171	0,272	0,259	0,215	0,259	0,3	0,273
Blasticidin hydrochloride	0,268	0,302	0,258	0,304	0,203	0,246	0,318	2,441	2,047	0,948	0,254	0,697	0,199	0,253	1,852	0,312	0,308	0,549	0,248	0,229	0,182	0,351	0,278	0,295	0,189	0,26	0,218	0,283	0,305	0,204	0,277	0,275	0,267	0,268	0,284	0,319
Thioridazine hydrochloride	0,282	0,298	0,247	0,288	0,202	0,256	0,295	2,067	2,23	2,131	0,27	1,624	0,232	0,262	2,126	0,313	0,307	2,061	0,256	0,236	0,195	0,349	0,328	0,31	0,189	0,262	0,248	0,282	0,304	0,267	0,279	0,289	0,292	0,282	0,294	0,316
Sodium benzoate	0,291	0,311	0,265	0,304	0,221	0,289	0,302	2,067	2,223	2,04	0,247	1,588	0,227	0,277	2,105	0,313	0,321	1,674	0,262	0,24	0,196	0,416	0,317	0,304	0,195	0,271	0,235	0,305	0,314	0,238	0,283	0,298	0,275	0,31	0,298	0,311
Chlortetracycline hydrochloride	0,346	0,343	0,307	0,323	0,268	0,31	0,338	2,095	1,399	1,387	0,336	0,741	0,264	0,352	1,37	0,403	0,398	0,724	0,4	0,3	0,268	0,402	0,31	0,402	0,225	0,368	0,281	0,336	0,408	0,282	0,33	0,442	0,324	0,328	0,416	0,382
Sodium metasillicate	0,259	0,31	0,257	0,294	0,222	0,257	0,275	2,11	2,077	0,491	0,296	0,615	0,242	0,295	2,028	0,338	0,33	0,822	0,311	0,253	0,19	0,351	0,289	0,308	0,204	0,324	0,242	0,29	0,323	0,231	0,28	0,324	0,255	0,289	0,295	0,34
Pentamidine Isethionate	0,406	0,323	0,288	0,296	0,227	0,283	0,306	2,026	2,076	1,438	0,258	1,515	0,309	0,264	2,161	0,29	0,308	1,669	0,301	0,295	0,36	0,422	0,268	0,341	0,2	0,262	0,286	0,356	0,349	0,271	0,378	0,298	0,357	0,3	0,336	0,346
6-Azauracil	0,329	0,293	0,247	0,287	0,196	0,246	0,362	2,252	2,261	1,953	0,247	1,648	0,226	0,258	2,058	0,305	0,296	1,702	0,314	0,229	0,198	0,316	0,364	0,285	0,243	0,259	0,204	0,257	0,315	0,277	0,274	0,297	0,236	0,294	0,293	0,314
Potassium chromate	0,273	0,283	0,258	0,369	0,228	0,257	0,268	1,788	1,405	1,855	0,246	1,524	0,246	0,269	1,334	0,329	0,319	1,701	0,27	0,267	0,202	0,34	0,248	0,291	0,205	0,323	0,23	0,286	0,334	0,2	0,28	0,325	0,24	0,304	0,312	0,338
Thialysine	0,319	0,27	0,258	0,279	0,221	0,26	0,306	2,185	2,001	1,411	0,226	1,519	0,199	0,232	1,914	0,284	0,275	1,253	0,266	0,217	0,189	0,335	0,229	0,231	0,181	0,246	0,268	0,286	0,309	0,25	0,317	0,331	0,253	0,291	0,312	0,313
Berberine	0,338	0,311	0,349	0,305	0,286	0,284	0,286	2,217	2,146	2	0,354	1,689	0,309	0,314	2,081	0,519	0,449	1,49	0,653	0,313	0,256	0,356	0,271	0,312	0,281	0,306	0,437	0,335	0,439	0,281	0,426	0,432	0,35	0,447	0,253	0,389
EGTA	0,309	0,322	0,311	0,369	0,3	0,32	0,266	2,393	2,282	2,175	0,305	1,884	0,261	0,33	2,248	0,375	0,393	2,052	0,435	0,32	0,221	0,402	0,273	0,342	0,218	0,413	0,277	0,36	0,392	0,319	0,33	0,455	0,319	0,351	0,34	0,46
Sodium pyrophosphate decahydrate	0,36	0,374	0,338	0,395	0,303	0,368	0,286	2,196	2,362	0,951	0,327	1,296	0,321	0,327	2,277	0,365	0,368	1,028	0,433	0,317	0,263	0,426	0,326	0,35	0,277	0,385	0,341	0,369	0,404	0,273	0,363	0,447	0,332	0,353	0,382	0,53
Isoniazid	0,392	0,269	0,238	0,282	0,203	0,258	0,267	1,59	1,545	0,757	0,215	0,514	0,178	0,238	1,692	0,281	0,265	0,551	0,382	0,216	0,184	0,319	0,261	0,295	0,182	0,239	0,219	0,254	0,277	0,144	0,258	0,251	0,207	0,28	0,271	0,254
Methyl viologen dichloride hydrate	0,289	0,354	0,296	0,276	0,221	0,318	0,302	1,714	1,305	0,704	0,297	0,646	0,321	0,314	1,203	0,4	0,333	0,48	0,289	0,295	0,26	0,374	0,341	0,29	0,233	0,363	0,283	0,32	0,398	0,334	0,305	0,446	0,292	0,291	0,369	0,347
Sodium fluoride	0,36	0,278	0,308	0,301	0,241	0,268	0,298	1,58	1,576	0,885	0,215	1,411	0,188	0,254	1,709	0,293	0,288	1,02	0,362	0,225	0,172	0,324	0,235	0,155	0,143	0,234	0,291	0,347	0,319	0,186	0,32	0,26	0,234	0,288	0,322	0,181
Cisplatin	0,675	0,288	0,263	0,3	0,209	0,253	0,297	1,394	1,447	0,69	0,244	0,628	0,219	0,246	1,416	0,295	0,283	0,568	0,59	0,236	0,186	0,33	0,257	0,306	0,196	0,266	0,242	0,308	0,319	0,186	0,271	0,274	0,248	0,316	0,301	0,305
Aluminium sulphate	0,346	0,39	0,292	0,337	0,245	0,291	0,27	1,537	1,393	0,712	0,323	0,781	0,253	0,358	1,464	0,317	0,375	0,474	0,336	0,261	0,252	0,44	0,329	0,304	0,284	0,292	0,286	0,317	0,347	0,339	0,367	0,317	0,26	0,325	0,376	0,341
Fluconazole	0,379	0,27	0,279	0,265	0,186	0,272	0,441	0,908	1,181	0,649	0,206	0,989	0,194	0,278	1,264	0,282	0,263	0,522	0,475	0,253	0,169	0,3	0,277	0,185	0,139	0,206	0,249	0,315	0,322	0,151	0,283	0,282	0,216	0,288	0,324	0,269
Propiconazole	0,328	0,294	0,265	0,33	0,215	0,311	0,277	1,656	1,189	1,035	0,221	0,759	0,209	0,253	1,256	0,286	0,278	1,098	0,471	0,227	0,189	0,315	0,243	0,271	0,194	0,258	0,227	0,276	0,295	0,178	0,262	0,271	0,216	0,279	0,275	0,276
Tamoxifen	0,33	0,298	0,262	0,328	0,218	0,276	0,297	1,497	1,185	0,641	0,222	0,494	0,225	0,25	1,301	0,28	0,287	0,505	0,486	0,248	0,192	0,31	0,255	0,287	0,175	0,258	0,244	0,267	0,306	0,191	0,267	0,272	0,204	0,273	0,264	0,261

Figure S3. Absorbance readings taken at 750nm after 48hr of incubation of isolates in a growth medium incorporated with the 24 compounds. Red indicates susceptibility and yellow indicates the resistance of isolates to the potential antifungal compounds.

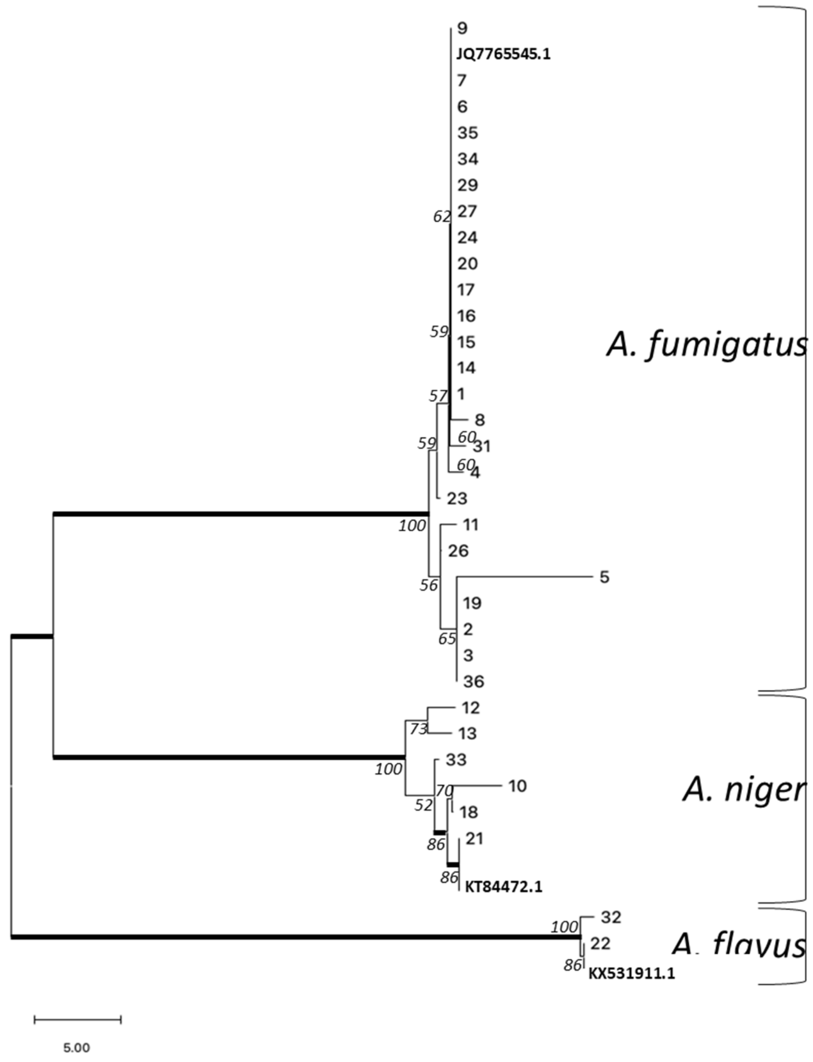

Figure S4. Phylogenic tree showing isolates of Aspergillus species. The sequences were aligned and the tree was done using Neighbour-Joining analysis and visualised on Treebase. dark lines that the branches with more than 75% support with UPGMA to the NJ tree (1000 bootstrap).
